# Structure of *Plasmodium vivaxN*-myristoyltransferase with inhibitor IMP-1088: exploring an NMT inhibitor for antimalarial therapy

**DOI:** 10.1107/S2053230X24011348

**Published:** 2025-01-01

**Authors:** Alex Mendez, Cydni Bolling, Shane Taylor, Stanley Makumire, Bart Staker, Alexandra Reers, Brad Hammerson, Stephen J. Mayclin, Jan Abendroth, Donald D. Lorimer, Thomas E. Edwards, Edward W. Tate, Sandhya Subramanian, Andrew S. Bell, Peter J. Myler, Oluwatoyin A. Asojo, Graham Chakafana

**Affiliations:** a Chemistry and Biochemistry Department Hampton University 200 William R. Harvey Way Hampton VA23668 USA; b Structural Biology Research Unit, Faculty of Biochemistry and Molecular Medicine University of Oulu Aapistie 7C 90220Oulu Finland; c Center for Global Infectious Disease Research Seattle Children’s Research Institute 307 Westlake Avenue North, Suite 500 Seattle WA98109 USA; d Seattle Structural Genomics Center for Infectious Diseases, Seattle, Washington, USA; e UCB BioSciences, Bainbridge Island, WA98110, USA; f Imperial College London South Kensington Campus LondonSW7 2AZ United Kingdom; g Myricx Pharma, 125 Wood Street, LondonEC2V 7AN, United Kingdom; h Dartmouth Cancer Center, One Medical Center Drive, Lebanon, NH03756, USA; University of York, United Kingdom

**Keywords:** *N*-myristoyltransferases, *Plasmodium vivax*, inhibitor complexes, drug repurposing, malaria

## Abstract

*Plasmodium vivax*, a significant contributor to global malaria cases, poses an escalating health burden on a substantial portion of the world’s population. The increasing spread of *P. vivax* because of climate change underscores the development of new and rational drug-discovery approaches. The Seattle Structural Genomics Center for Infectious Diseases is taking a structure-based approach by investigating essential enzymes such as *N*-myristoyltransferase (NMT). *P. vivaxN*-myristoyltransferase (*Pv*NMT) is a promising target for the development of novel malaria treatments unlike current drugs, which target only the erythrocytic stages of the parasite. Here, the 1.8 Å resolution ternary structure of *Pv*NMT in complex with myristoyl-CoA and IMP-1088, a validated NMT inhibitor, is reported. IMP-1088 is a validated nonpeptidic inhibitor and a ternary complex structure with human NMT has previously been reported. IMP-1088 binds similarly to *Pv*NMT as to human NMT.

## Abstract 2

The 1.8 Å resolution ternary structure of *P. vivaxN*-myristoyltransferase in complex with IMP-1088 and myristoyl-CoA is presented. IMP-1088 is an *N*-myristoyltransferase inhibitor from a family of inhibitors that are under investigation as antimalarials.

## 1. Introduction

*Plasmodium vivax* is responsible for the most widespread form of malaria and approximately 2.5 billion people, or over one-third of the world’s population, are at risk of *P. vivax* infection (Battle *et al.*, 2019 ▸). In humans, *P. vivax* can enter a dormant liver phase, which allows it to survive in various climates, including tropical and temperate regions, and contributes to its extensive geographical prevalence (Battle *et al.*, 2019 ▸). *P. vivax* infection significantly impacts the quality of life of infected individuals, causing cyclical episodes of fever and weakness, representing a substantial burden in endemic countries due to treatment costs and productivity loss. *P. vivax* can persist in human hosts as hypnozoites in the liver that can cause relapses that can extend over several months or years (Flannery *et al.*, 2022 ▸). Curing vivax malaria requires antimalarial drugs that are effective against both the blood and liver stages. Unfortunately, the sole licensed antimalarial with *P. vivax* activity, primaquine, has the drawback of inducing severe hemolysis in those with glucose-6-phosphate dehydrogenase (G6PD) deficiency, representing approximately 15% of the population in *P. vivax* endemic regions (Douglas *et al.*, 2023 ▸).

The Seattle Structural Genomics Center for Infectious Diseases and collaborators are investigating rational malaria therapeutics discovery targeting essential proteins (Vijayan *et al.*, 2021 ▸).

These efforts identified *P. vivaxN*-myristoyltransferase (*Pv*NMT) inhibitors that overcome drug resistance (Schlott *et al.*, 2019 ▸). *Pv*NMT is an essential enzyme that catalyzes a post-translational modification (myristoylation) through transfer of the lipid myristate from myristoyl coenzyme A (Myr-CoA) to the N-terminal glycine residues of proteins (Selvakumar *et al.*, 2011 ▸; Udenwobele *et al.*, 2017 ▸; McIlhinney, 1989 ▸). *Pv*NMT catalyzes the myristoylation of substrate proteins that modulate crucial parasite cellular processes such as membrane association, protein–protein interactions, stability, turnover and signal transduction (Schlott *et al.*, 2021 ▸; Selvakumar *et al.*, 2011 ▸). Examples of plasmodial proteins that are myristoylated by NMT include glideosome-associated protein 45 (GAP45), which cannot perform its erythrocyte-invasion roles unless it is myristoylated (Schlott *et al.*, 2021 ▸). Myristoylation of erythrocyte-binding antigen 175 (EBA-175) is required for *P. vivax* to invade erythrocytes (Bouyssou *et al.*, 2023 ▸). Plasmodial exported protein 1 (EXP-1) and early transcribed membrane protein 11.2 (ETMP-11.2) must be myristoylated for parasites to exit the red blood cell (Cheng *et al.*, 2015 ▸). Consequently, *Pv*NMT inhibition significantly affects parasite development and survival (Garcia *et al.*, 2022 ▸; Nicolau *et al.*, 2023 ▸; Rodríguez-Hernández *et al.*, 2023 ▸). Plasmodial adenylate kinases 2 are liver-stage proteins that must be myristoylated (Rodríguez-Hernández *et al.*, 2023 ▸).

NMTs have been validated as targets for multiple parasitic diseases, including trypanosomiasis and leishmaniases (Corpas-Lopez *et al.*, 2019 ▸; Wright *et al.*, 2014 ▸; Frearson *et al.*, 2010 ▸; Rodríguez-Hernández *et al.*, 2023 ▸; Harupa *et al.*, 2020 ▸). NMTs are promising drug targets for malaria and other diseases (Priyamvada *et al.*, 2022 ▸; Garcia *et al.*, 2022 ▸; Goncalves *et al.*, 2017 ▸; Javid *et al.*, 2023 ▸; Rackham *et al.*, 2014 ▸; Rodríguez-Hernández *et al.*, 2023 ▸; Bolling *et al.*, 2024 ▸; Bell *et al.*, 2012 ▸, 2020 ▸, 2022 ▸). The first reported family of NMT inhibitors was developed through rational design strategies utilizing peptide-mimicking substrates or nonhydrolyzable Myr-CoA analogs. Subsequently, novel families of NMT inhibitors have been identified through high-throughput screening (HTS) efforts (Goncalves *et al.*, 2017 ▸).

IMP-1088 is an effective antipicornaviral agent with selectivity and pharmacological activity against NMT (Mousnier *et al.*, 2018 ▸; Wright *et al.*, 2014 ▸). IMP-1088 also effectively inhibits the production of infectious rhinovirus virions by blocking the *N*-myristoylation of rhinovirus VP0 (Mousnier *et al.*, 2018 ▸). Other IMP-1088 chemotypes have been developed against NMT to treat multiple diseases, with recent efforts focusing on the development of novel *Pv*NMT inhibitors as antimalarials (Bell *et al.*, 2012 ▸; Rodríguez-Hernández *et al.*, 2023 ▸; Schlott *et al.*, 2018 ▸). Here, we present the structure of IMP-1088 in complex with *Pv*NMT. Comparing the reported structure with that of human NMT in complex with IMP-1088 (PDB entry 5mu6; Mousnier *et al.*, 2018 ▸) offers insights into repurposing this family of compounds as antimalarials.

## 2. Materials and methods

### 2.1. Macromolecule production

A codon-optimized gene (*Pv*NMT; UniProt A0A1G4H3M1), encoding amino acids 27–410, was synthesized by GenScript with a 3C protease-cleavable hexahistidine tag (MGSSHHHHHHSAALEVLFQGP-ORF). Plasmid DNA was transformed into chemically competent *Escherichia coli* BL21(DE3) cells (Table 1 ▸). The plasmid containing His-*Pv*NMT was tested for expression, and 2 l of culture was grown using auto-induction medium (Studier, 2005 ▸) in a LEX Bio­reactor (Epiphyte Three) as described previously (Serbzhinskiy *et al.*, 2015 ▸). The expression clone can be requested online at https://www.ssgcid.org/available-materials/expression-clones/.

*Pv*NMT was purified in two steps: an immobilized metal (Ni^2+^) affinity chromatography (IMAC) step and size-exclusion chromatography (SEC) on an AKTApurifier 10 (GE Healthcare, now Cytiva) using automated IMAC and SEC programs (Serbzhinskiy *et al.*, 2015 ▸). Briefly, thawed bacterial pellets (25 g) were lysed by sonication in 200 ml lysis buffer [25 m*M* HEPES pH 7.0, 500 m*M* NaCl, 5%(*v*/*v*) glycerol, 0.5%(*w*/*v*) CHAPS, 30 m*M* imidazole, 10 m*M* MgCl_2_, 1 m*M*TCEP and five tablets of protease-inhibitor cocktail (cOmplete Mini, EDTA-free Roche, Basel, Switzerland)]. After sonication, the crude lysate was treated with 20 µl (25 U ml^−1^) of Benzonase by incubating and mixing at room temperature for 45 min. The lysate was clarified by centrifugation at 5000*g* for 1 h at 277 K using a refrigerated Sorvall centrifuge (Thermo Scientific). The clarified supernatant was then passed over a 5 ml Ni–NTA HisTrap FF column (GE Healthcare, now Cytiva) which had been pre-equilibrated with loading buffer [25 m*M* HEPES pH 7.0, 500 m*M* NaCl, 5%(*v*/*v*) glycerol, 30 m*M* imidazole, 1 m*M* TCEP, 0.025%(*w*/*v*) sodium azide]. The column was washed with 20 column volumes (CV) of loading buffer and eluted with elution buffer [25 m*M* HEPES pH 7.0, 500 m*M* NaCl, 5%(*v*/*v*) glycerol, 30 m*M* imidazole, 1 m*M* TCEP, 0.025%(*w*/*v*) sodium azide, 250 m*M* imidazole] over a 7 CV linear gradient. Peak fractions were pooled, concentrated to 5 ml and loaded onto a Superdex 75 26/60 column (GE Biosciences) equilibrated with running buffer (20 m*M* HEPES pH 7.0, 300 m*M* NaCl, 5% glycerol, 1 m*M* TCEP). *Pv*NMT eluted from the SEC column as a single, monodisperse symmetrical peak accounting for >90% of the protein product at a molecular mass of ∼40 kDa, suggesting purification as a monomer (based on a theoretical molecular weight of 47.1 kDa). The pure peak fractions were pooled and concentrated to 13.5 mg ml^−1^ using an Amicon purification system (Millipore). The purified protein was stored in 100 µl aliquots at 193 K and can be requested online at https://www.ssgcid.org/available-materials/ssgcid-proteins/.

### 2.2. Crystallization

*Pv*NMT was crystallized using sitting-drop vapor diffusion as described in Table 2 ▸. Crystals were harvested and cryoprotected with 20% ethylene glycol before data collection.

### 2.3. Data collection and processing

Data were collected at 100 K as detailed in Table 3 ▸. Data were integrated using *XDS* (Kabsch, 2010 ▸) and reduced with *XSCALE* (Kabsch, 2010 ▸).

### 2.4. Structure solution and refinement

The structure was determined by molecular replacement with *MOLREP* from the *CCP*4 suite of programs (Collaborative Computational Project, Number 4, 1994 ▸; Krissinel *et al.*, 2004 ▸; Winn *et al.*, 2011 ▸; Agirre *et al.*, 2023 ▸) using PDB entry 4b14 (with inhibitors and waters removed) as the search model (Yu *et al.*, 2012 ▸). The structure was refined using *Phenix* (Liebschner *et al.*, 2019 ▸). The refined structure quality was assessed using *MolProbity* (Williams *et al.*, 2018 ▸). Refinement statistics are listed in Table 4 ▸. The coordinates and structure factors have been deposited with the Worldwide Protein Data Bank (wwPDB) as PDB entry 5v0w. Omit electron-density maps reveal ordered electron density for all of the ligands (Supplementary Fig. S1). The ligands and waters were also checked with the *CheckMyBlob* server (Kowiel *et al.*, 2019 ▸; https://checkmyblob.bioreproducibility.org/server/).

## 3. Results and discussion

**Figure 1:**
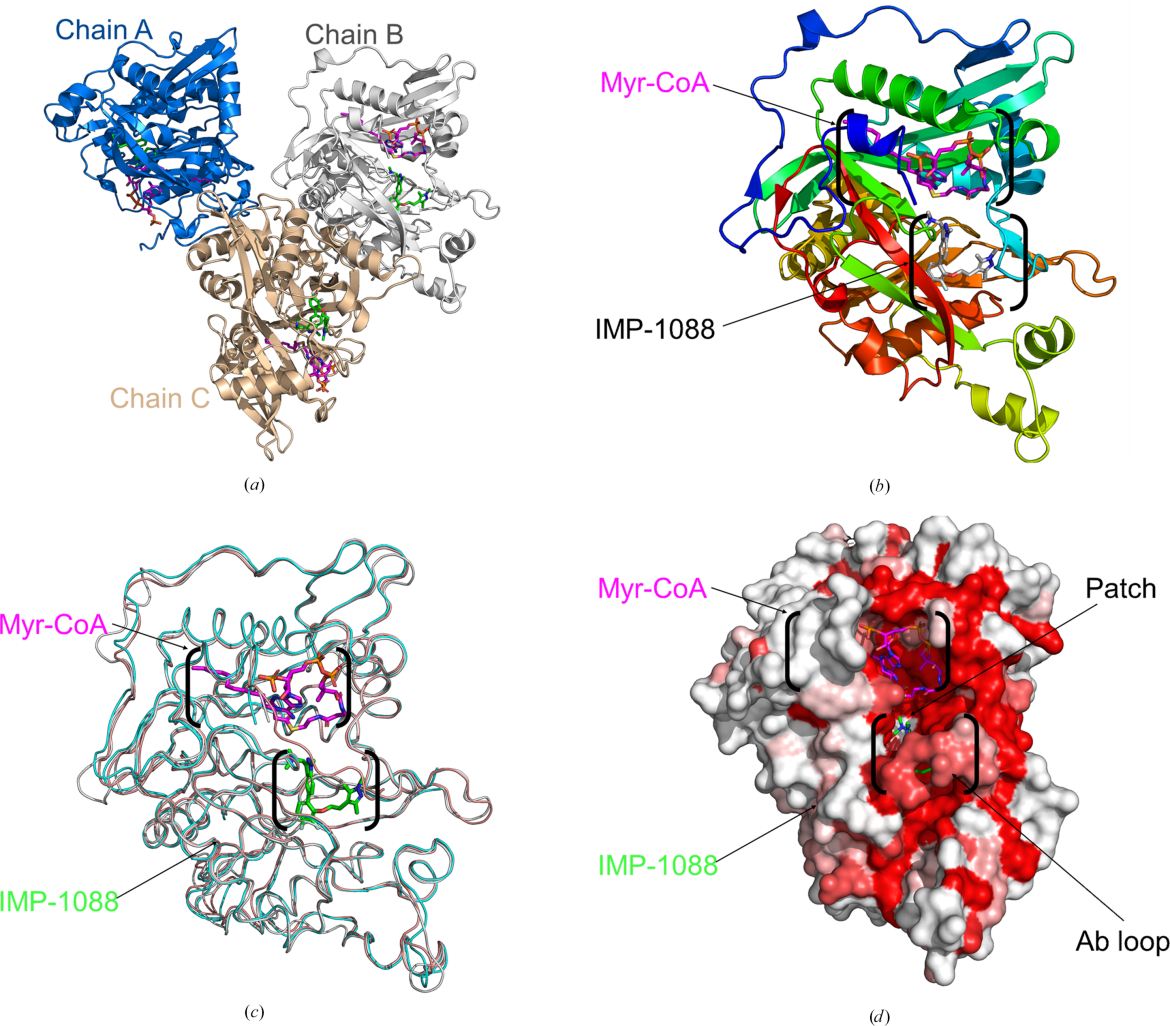
Ternary structure of *Pv*NMT with Myr-CoA and the inhibitor IMP-1088. (*a*) There are three *Pv*NMT monomers in the asymmetric unit: *A* (marine), *B* (gray) and *C* (light brown). Each has a bound Myr-CoA (magenta) and IMP-1088 inhibitor (green). (*b*) Superposed monomers are almost identical, with an r.m.s.d. of ∼0.10 Å on C^α^ atoms. Each monomer is colored in a rainbow from blue at the N-terminus to red at the C-terminus. Myr-CoA is shown as magenta sticks, while the inhibitor IMP-1088 is shown as green sticks. (*c*) Superposed monomers of *Pv*NMT (PDB entry 5v0w, gray), *Hs*NMT1 (PDB entry 5mu6, pink) and *Hs*NMT2 (PDB entry 4c2x, cyan). Myr-CoA is shown as magenta sticks, while the inhibitor IMP-1088 is shown as green sticks. (*d*) Solvent-accessible surface area of *Pv*NMT colored by sequence conservation, with red indicating identical residues. The peptide/substrate-binding and myristoyl-binding cavities are shown in black parentheses.

The ternary structure of *Hs*NMT1 bound to Myr-CoA and IMP-1088 was previously reported as PDB entry 5mu6 (Mousnier *et al.*, 2018 ▸). Our reported ternary complex of *Pv*NMT, Myr-CoA and IMP-1088 allows structure–function comparison of host and parasite inhibition by the same non­peptidic inhibitor. The ternary complex of *Pv*NMT, Myr-CoA and the nonpeptidic inhibitor IMP-1088 was determined at a resolution of 1.8 Å (Table 3 ▸). The asymmetric unit contains three monomers (Fig. 1 ▸*a*). The three almost identical monomers adopt the prototypical NMT topology (Dian *et al.*, 2020 ▸), with a compact, spherical configuration comprising 15 α-helices and 19 β-sheets (Figs. 1 ▸*a* and 1 ▸*b*). Two monomers (chains *A* and *B*) have 385 amino-acid residues (residues 26–10) and the third (chain *C*) has 377 residues.

Each monomer has two N-terminal binding cavities: the peptide/substrate-binding cavity containing the inhibitor IMP-1088 and the myristoyl-binding cavity containing Myr-CoA (Fig. 1 ▸*b*). Consistent with other *Pv*NMT structures, a central core with an internal pseudo-twofold symmetry axis formed by the N-terminal and C-terminal halves shapes the structure of the peptide-binding site (Goncalves *et al.*, 2017 ▸; Rodríguez-Hernández *et al.*, 2023 ▸; Rudnick *et al.*, 1993 ▸; Spassov *et al.*, 2023 ▸; Bolling *et al.*, 2024 ▸). All loops that are near or interacting with both binding cavities are ordered in all three monomers, notably the *ab* loop, which forms a lid that embraces the inhibitor within the peptide/substrate-binding cavity (Fig. 1 ▸*d*).

**Figure 2:**
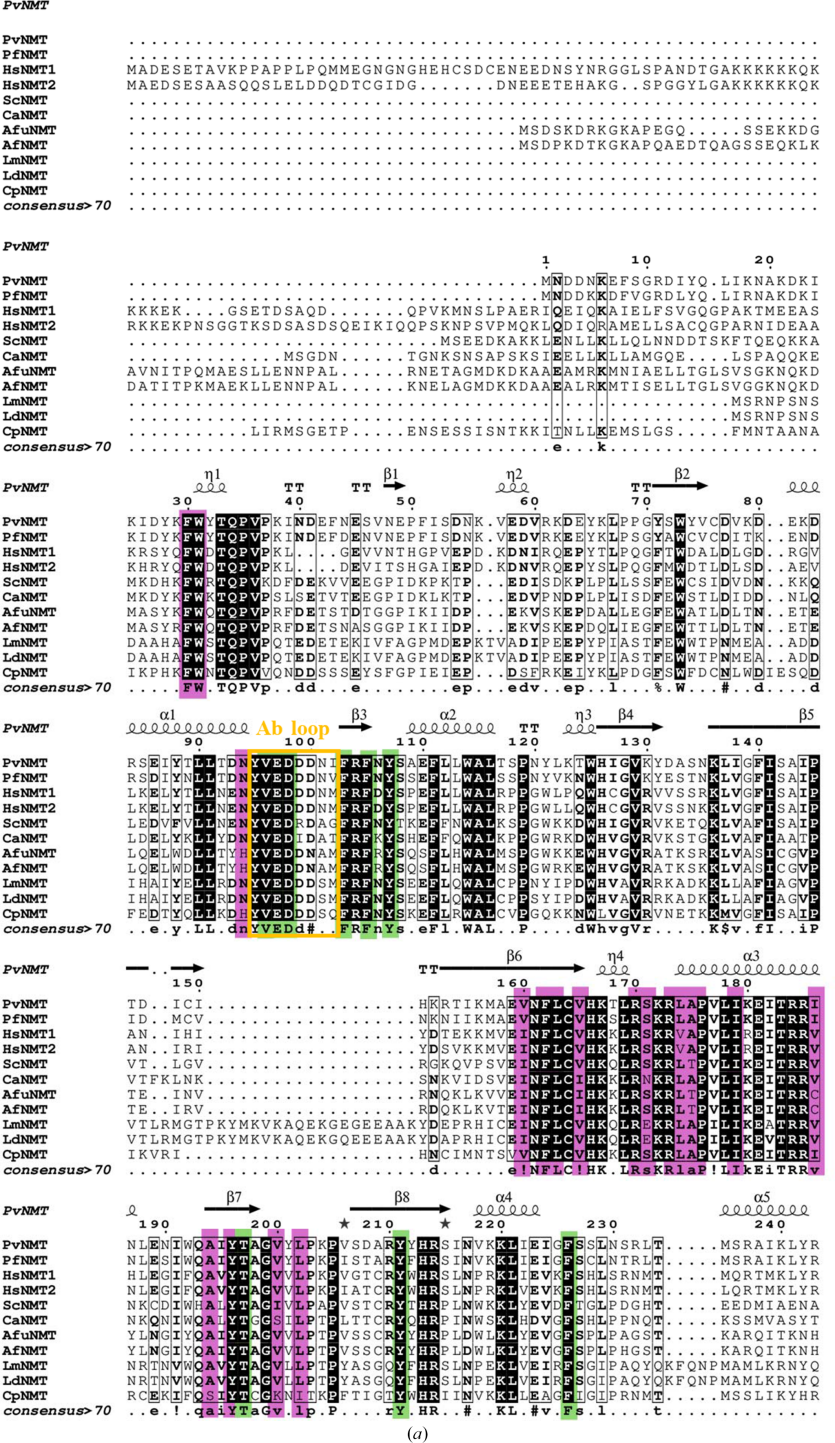

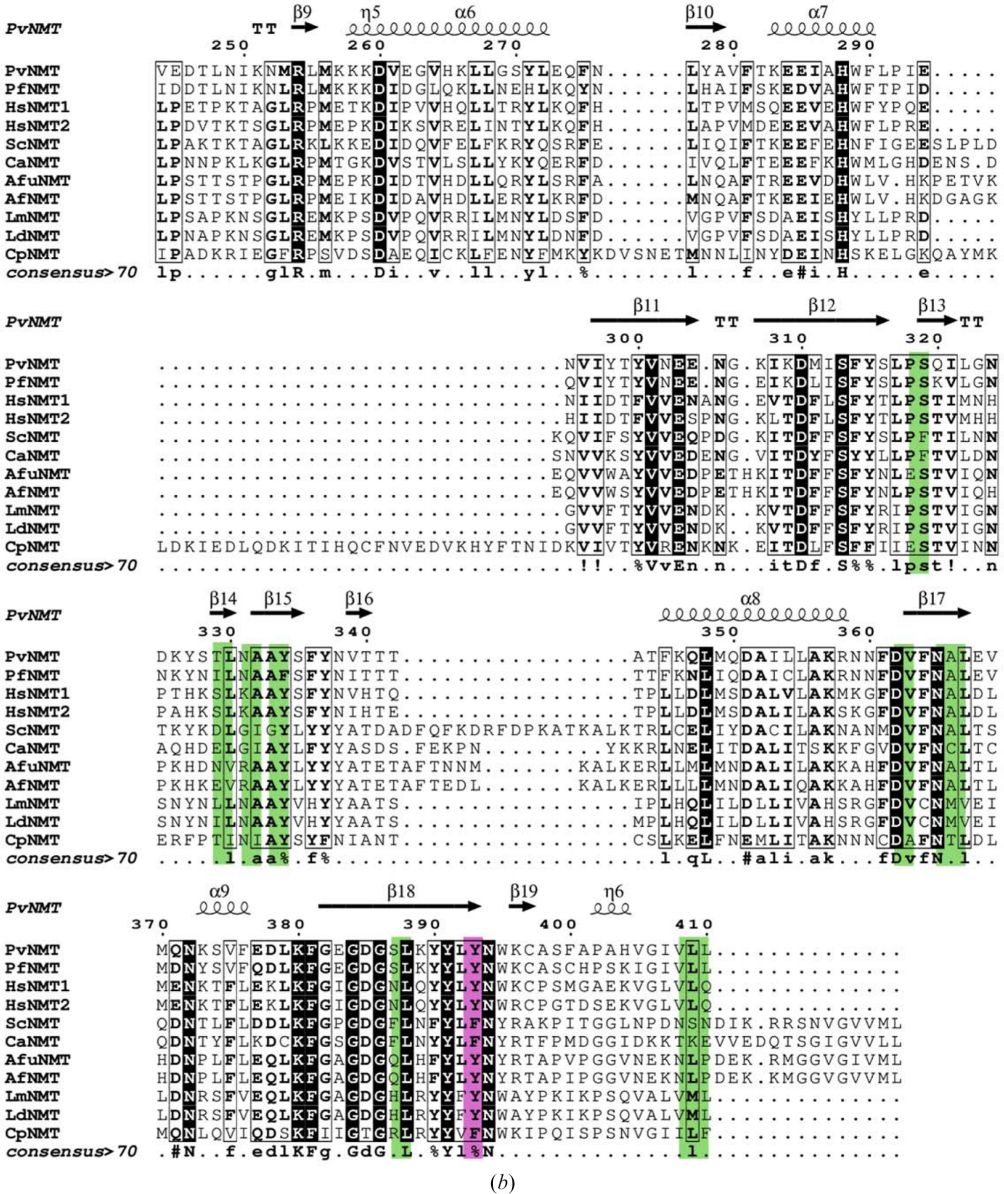
(*a*) The alignment shows residue conservation between NMTs from different organisms. Residues within the cofactor-binding pocket are shown in purple, while those in the substrate-binding pocket are shown in green. The secondary-structure alignment is based on our reported structure. (*b*) The residues located in the C-termini of different NMTs. The following NMTs are included in the alignment: *Pv*NMT, *P. falciparum* NMT, *Hs*NMT1, *Hs*NMT2, *Saccharomyces cerevisiae* NMT, *Candida albicans* NMT, *Aspergillus fumigatus* NMT, *A. flavus* NMT, *Leishmania major* NMT, *L. donovani* NMT and *Cryptosporidium parvum* NMT.

**Figure 3:**
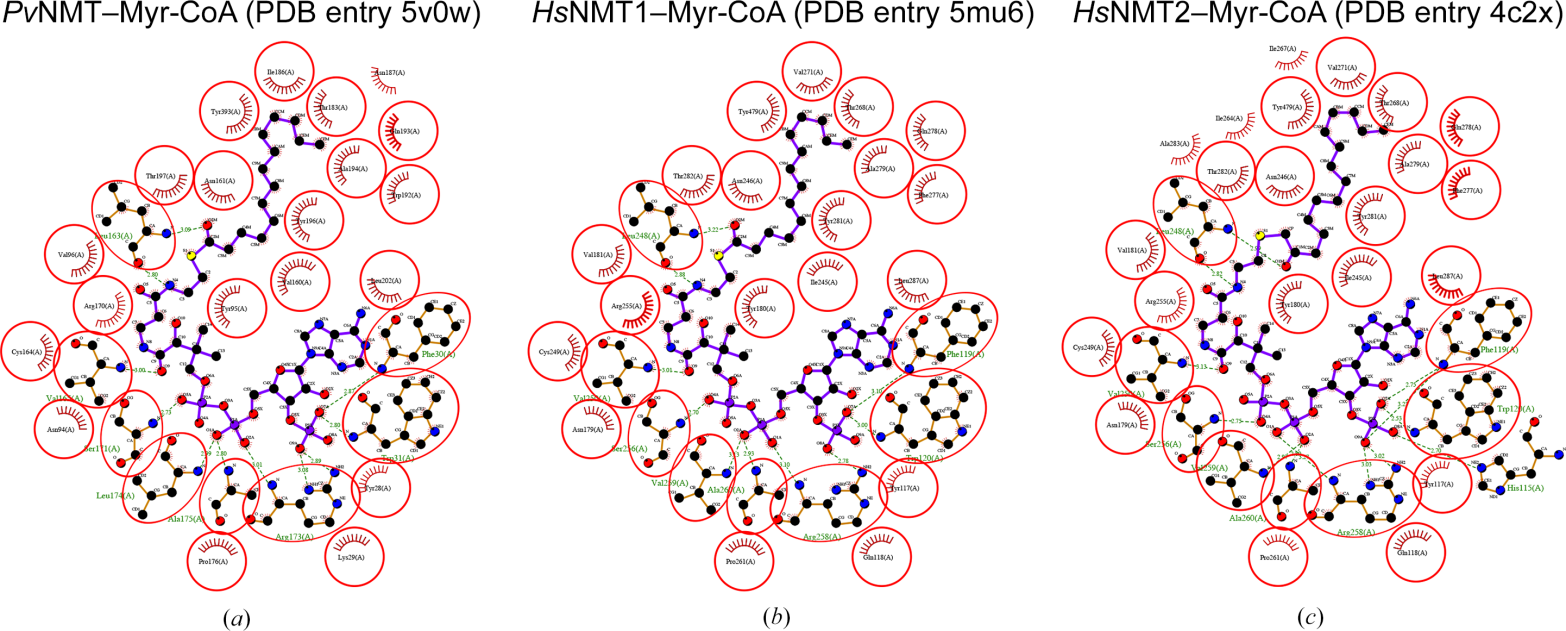
Myr-CoA binding by NMTs. Conserved amino-acid residues mediate Myr-CoA binding in (*a*) *Pv*NMT (PDB entry 5v0w), (*b*) *Hs*NMT1 (PDB entry 5mu6) and (*c*) *Hs*NMT2 (PDB entry 4c2x). This figure and other ligand-interaction figures were generated with *LigPlus *(https://www.ebi.ac.uk/thornton-srv/software/LigPlus/).

The top 82 closest structural neighbors of the reported structure were identified by *PDBeFold* (https://www.ebi.ac.uk/msd-srv/ssm/) analysis (Krissinel & Henrick, 2004 ▸) using a default threshold of 70% to be *Pv*NMT structures with various ligands. The next 63 are human NMT structures, followed by NMTs from other organisms (Supplementary Table S1). *ENDScript* analyses (Gouet *et al.*, 2003 ▸; Robert & Gouet, 2014 ▸) validate the *PDBeFold* results and reveal well conserved amino acids across the different NMTs (Supplementary Fig. S2). Structural and primary-sequence alignment reveals significant secondary-structure similarity between human and plasmodial NMTs (Fig. 2 ▸). Superposed ribbons also show the similarity in tertiary structure of human and plasmodial NMTs (Fig. 1 ▸*c*). A surface diagram of *Pv*NMT reveals that the regions with the highest similarity are near the interconnected Myr-CoA-binding and peptide-binding cavities, as shown in red in Fig. 1 ▸(*d*). Notably, the myristoyl-binding cavity is well conserved across NMTs (Fig. 1 ▸*d*). Myr-CoA binding is stabilized by a few positive charges in the mainly hydrophobic myristoyl-binding cavity (Harupa *et al.*, 2020 ▸; Rodríguez-Hernández *et al.*, 2023 ▸; Bolling *et al.*, 2024 ▸). *LigPlus* analysis (Laskowski & Swindells, 2011 ▸; Wallace *et al.*, 1995 ▸) shows that the amino acids interacting with Myr-CoA are almost identical in *Pv*NMT (PDB entry 5v0w) compared with *Hs*NMT1 (PDB entry 5mu6) and *Hs*NMT2 (PDB entry 4c2x) (Fig. 3 ▸).

**Figure 4:**
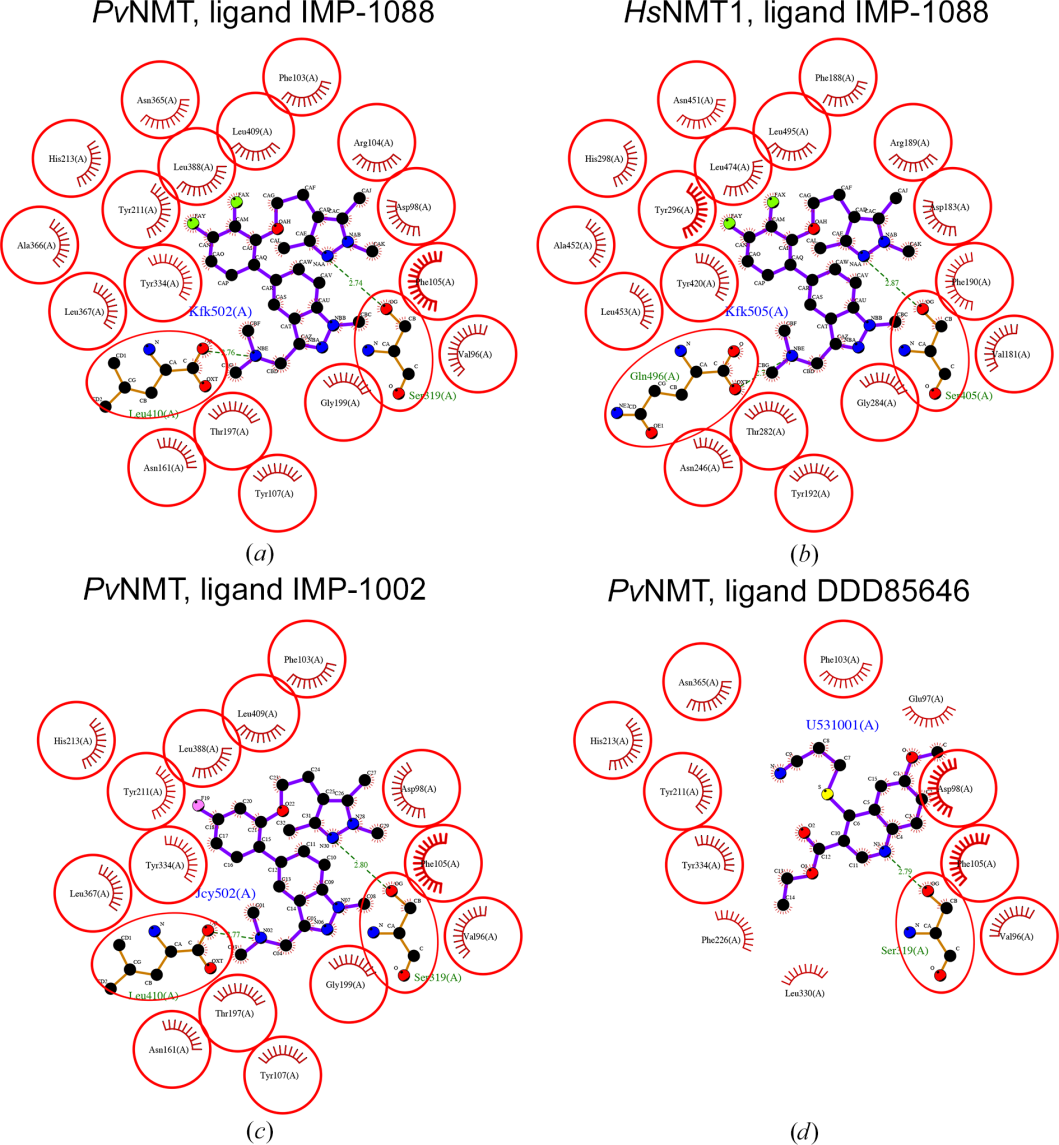
Comparison of inhibitor binding by NMTs. *Pv*NMT interacts with IMP-1088 (PDB entry 5v0w) (*a*) with similar amino acids as *Hs*NMT1 (PDB entry 5mu6) (*b*). Inhibitor IMP-1002 interacts with fewer amino acids on *Pv*NMT (PDB entry 6mb1) (*c*), as does inhibitor DDD85646 with *Pv*NMT (PDB entry 5g1z) (*d*).

IMP-1088 binds to a predominantly hydrophobic peptide/substrate-binding cavity stabilized by several hydrogen bonds and salt bridges (Fig. 4 ▸*a*). The peptide/substrate-binding cavity is less well conserved across NMTs (Bolling *et al.*, 2024 ▸), as indicated by the white patch in Fig. 1 ▸(*d*). The amino-acid residues interacting with IMP-1088 are almost identical in the *Pv*NMT (PDB entry 5v0w) and *Hs*NMT1 (PDB entry 5mu6) structures. Notably, the serine mediating a hydrogen bond involved in inhibitor binding is conserved, as are most residues involved in IMP-1088 binding (Figs. 4 ▸*a* and 4 ▸*b*). Nonetheless, while *Pv*NMT interacts with IMP-1088 through a leucine residue (Leu410), *Hs*NMT forms contacts with the compound via a glutamine residue (Gln496) (Figs. 4 ▸*a* and 4 ▸*b*).

IMP-series inhibitors generally exhibit excellent efficacy against *P. vivax* (Mousnier *et al.*, 2018 ▸). For example, IMP-1031, an analog of IMP-1088, had an IC_50_ value of approximately 200 p*M* in a *P. berghei* liver-stage assay (Bell *et al.*, 2012 ▸, 2020 ▸, 2022 ▸). The comparison of complex structures of *Pv*NMT and promising IMP-series inhibitors reveals similar interactions (Fig. 4 ▸). IMP-1002, an analog of IMP-1088 discovered through a fragment-reconstruction approach based on hits from screens against *Pv*NMT and *P. falciparum* NMT (Mousnier *et al.*, 2018 ▸; Schlott *et al.*, 2019 ▸), binds similarly to IMP-1088. Interestingly, IMP-1002 exhibits a fourfold higher potency in killing parasites than the most potent previously reported *Pv*NMT inhibitor, DDD85646 (Wright *et al.*, 2014 ▸). *LigPlus* analysis of the *Pv*NMT structures reveals that DDD85646 (PDB entry 5g1z) interacts with fewer amino-acid residues than IMP-1002 (PDB entry 6mb1) and IMP-1088 (PDB entry 5v0w) (Figs. 4 ▸*c* and 4 ▸*d*, Table 5 ▸).

**Figure 5:**
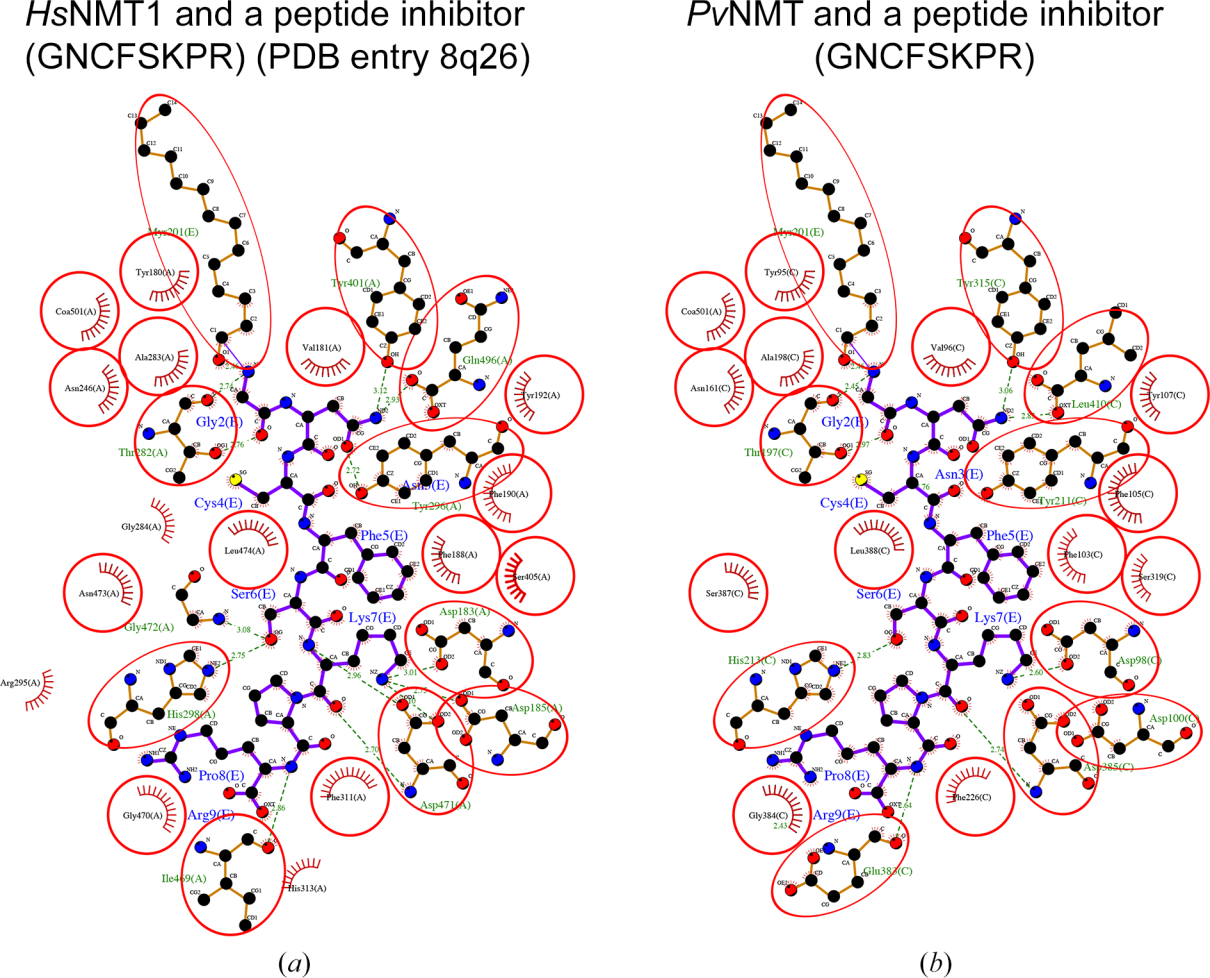
Interactions in the peptide/substrate-binding cavity. (*a*) Interactions between *Hs*NMT1 and a peptide inhibitor (GNCFSKPR; PDB entry 8q26). (*b*) The modeled superposed *Pv*NMT structure (starting from PDB entry 5v0w with ligands removed) shows conserved interactions with the peptide inhibitor.

The structure of the complex of *Hs*NMT1 with an inhibitor peptide (GNCFSKPR) and Myr-CoA (PDB entry 8q26) was released in August 2024 (Rivière *et al.*, 2024 ▸). This structure allows the entire peptide-binding cavity of *Hs*NMT1 to be probed, revealing the amino-acid residues involved in peptide binding (Fig. 5 ▸). *LigPlus* analysis after alignment of the peptide (GNCFSKPR) inhibitor with *Pv*NMT reveals a similar network of amino-acid interactions within well conserved substrate/peptide-binding cavities (Fig. 5 ▸). Substrate-binding specificity is ensured via the preferential binding of glycine residues by the myristoyl-binding cavity (Harupa *et al.*, 2020 ▸).

## 4. Conclusions

The ternary structure of *P. vivaxN*-myristoyltransferase (*Pv*NMT) with IMP-1088 and Myr-CoA is presented. Ongoing efforts to develop IMP-1088-like compounds as antimalarials include testing the inhibitory activity of IMP-1088 against *Pv*NMT.

## Supplementary Material

PDB reference: *P. vivaxN*-myristoyltransferase, complex with myristoyl-CoA and IMP-1088, 5v0w

Supplementary figures. DOI: 10.1107/S2053230X24011348/ir5035sup1.pdf

## Figures and Tables

**Table 1:** Macromolecule-production information

Source organism	*Plasmodium vivax* Salvador I
DNA source	Synthetic, GenScript
Cloning vector	pET-11a
Expression vector	PCR-amplified plasmid DNA
Expression host	*Escherichia coli* BL21(DE3)R3 Rosetta
Complete amino-acid sequence of the construct produced	MGSSHHHHHHSAALEVLFQGPDYKFWYTQPVPKINDEFNESVNEPFISDNKVEDVRKDEYKLPPGYSWYVCDVKDEKDRSEIYTLLTDNYVEDDDNIFRFNYSAEFLLWALTSPNYLKTWHIGVKYDASNKLIGFISAIPTDICIHKRTIKMAEVNFLCVHKTLRSKRLAPVLIKEITRRINLENIWQAIYTAGVYLPKPVSDARYYHRSINVKKLIEIGFSSLNSRLTMSRAIKLYRVEDTLNIKNMRLMKKKDVEGVHKLLGSYLEQFNLYAVFTKEEIAHWFLPIENVIYTYVNEENGKIKDMISFYSLPSQILGNDKYSTLNAAYSFYNVTTTATFKQLMQDAILLAKRNNFDVFNALEVMQNKSVFEDLKFGEGDGSLKYYLYNWKCASFAPAHVGIVLL

**Table 2:** Crystallization

Method	Vapor diffusion, sitting drop
Plate type	Tray 101-d6, 96-well plates
Temperature (K)	290
Protein concentration (mg ml^−1^)	14.88
Buffer composition of protein solution	20 m*M* HEPES pH 7.0, 300 m*M* NaCl, 5%(*v*/*v*) glycerol, 1 m*M* TCEP, 0.5 m*M* IMP-1088, 0.5 m*M* Myr-CoA
Composition of reservoir solution	27% PEG 3350, 200 m*M* ammonium sulfate, 100 m*M* bis-Tris pH 6.0
Volume and ratio of drop	0.4 µl, 1:1
Volume of reservoir (µl)	80

**Table 3:** Data collection and processing Values in parentheses are for the outer shell.

Diffraction source	Beamline 08ID-1, Canadian Light Source
Wavelength (Å)	0.97949
Temperature (K)	100
Detector	Rayonix MX-300 CCD
Space group	*P*2_1_2_1_2_1_
*a*, *b*, *c* (Å)	57.32, 119.13, 176.61
α, β, γ (°)	90, 90, 90
Resolution range (Å)	50–1.80 (1.85–1.80)
No. of unique reflections	112736
Completeness (%)	99.9 (99.4)
Multiplicity	7.2 (6.2)
〈*I*/σ(*I*)〉	15.21 (2.98)
*R*_r.i.m._	0.115 (0.562)
Overall *B* factor from Wilson plot (Å^2^)	12.320

**Table 4:** Structure solution and refinement Values in parentheses are for the outer shell.

Resolution range (Å)	50.0–1.80 (1.84–1.80)
Completeness (%)	99.9
σ Cutoff	*F* > 1.35σ(*F*)
No. of reflections, working set	112717 (7250)
No. of reflections, test set	2031 (119)
Final *R*_cryst_	0.147 (0.227)
Final *R*_free_	0.184 (0.292)
No. of non-H atoms	
Protein	9405
Ion	60
Ligand	288
Solvent	1697
Total	11450
R.m.s. deviations	
Bond lengths (Å)	0.007
Angles (°)	0.935
Average *B* factors (Å^2^)	
Protein	14.5
Ion	53.4
Ligand (IMP-1088)	15.5
Ligand (myristoyl-CoA)	17.5
Water	27.8
Ramachandran plot	
Most favored (%)	97
Allowed (%)	3

**Table 5:** Residues involved in ligand binding

	PDB entry 5v0w	PDB entry 5mu6	PDB entry 6mb1	PDB entry 5g1z
Hydrogen-bond contacts	Ser319	Ser405	Ser319	Ser319
Non-hydrogen-bond contacts	Val96	Val181	Val96	Val96
	Asp98	Asp183	Asp98	Glu97
	Phe103	Phe188	Phe103	Asp98
	Arg104	Arg189	Phe105	Phe103
	Phe105	Phe190	Tyr107	Phe105
	Tyr107	Tyr192	Asn161	Tyr211
	Asn161	Asn246	Thr197	Phe226
	Thr197	Thr282	Gly199	Ser319
	Gly199	Gly284	Tyr211	Leu330
	Tyr211	Tyr296	Ser319	Tyr334
	His213	His298	Tyr334	Asn365
	Ser319	Ser405	Asn365	
	Tyr334	Tyr420	Ala366	
	Asn365	Asn451	Leu367	
	Ala366	Ala452	Leu388	
	Leu367	Leu453	Leu409	
	Leu388	Leu474	Leu410	
	Leu409	Leu495		
	Leu410	Gln496		
