# Supplementary material for: Structure of Plasmodium vivaxN-myristoyltransferase with inhibitor IMP-1088: exploring an NMT inhibitor for antimalarial therapy
Source: Acta Crystallogr F Struct Biol Commun. 2025 Jan 1;81(Pt 1):1–10. doi: 10.1107/S2053230X24011348 (PMC11701927; doi:10.1107/S2053230X24011348)
Supplement: Supplementary file 1 [file f-81-00001-sup1.pdf]

Acta Cryst F

STRUCTURAL BIOLOGY COMMUNICATIONS

Volume 80 (2024)

Supporting information for article:

Structure of *Plasmodium vivax N*-myristoyltransferase with inhibitor IMP-1088

Alex Mendez, Cydni Bolling, Shane Taylor, Stanley Makumire, Bart Staker, Alexandra Reers, Brad Hammerson, Stephen J. Mayclin, Jan Abendroth, Donald D. Lorimer, Thomas E. Edwards, Edward W. Tate, Sandhya Subramanian, Andrew S. Bell, Peter J. Myler, Oluwatoyin A. Asojo and Graham Chakafana

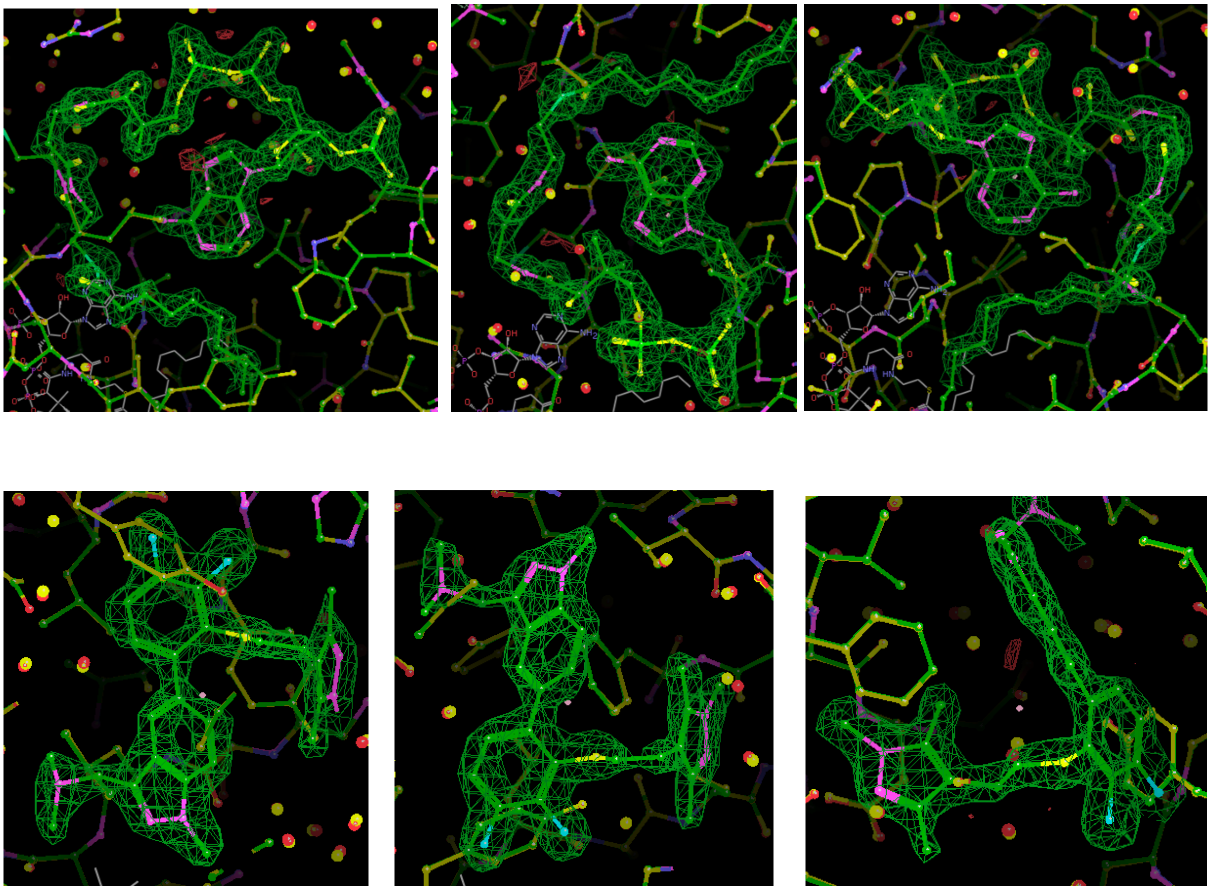

Figure S1 Difference (Fo-Fc) omit electron density maps (green mesh) contoured at 4.5 sigma revealed six large blobs that matched the superposed ligands (shown in stick). The top row shows the 3 Myr-CoA molecules, while the bottom row shows the 3 inhibitor IMP-1088 molecules.

Figure S2. ENDS CRIPT analysis reveals the nearest structural neighbors of *Pv*NMT (PDB entry 5V0W) and shows extensive sequence conservation across multiple organisms. Identical and conserved residues are highlighted in red and yellow, respectively. The different secondary structure elements shown are alpha helices (α), 3_{10}-helices (η), beta strands (β), and beta turns (TT).

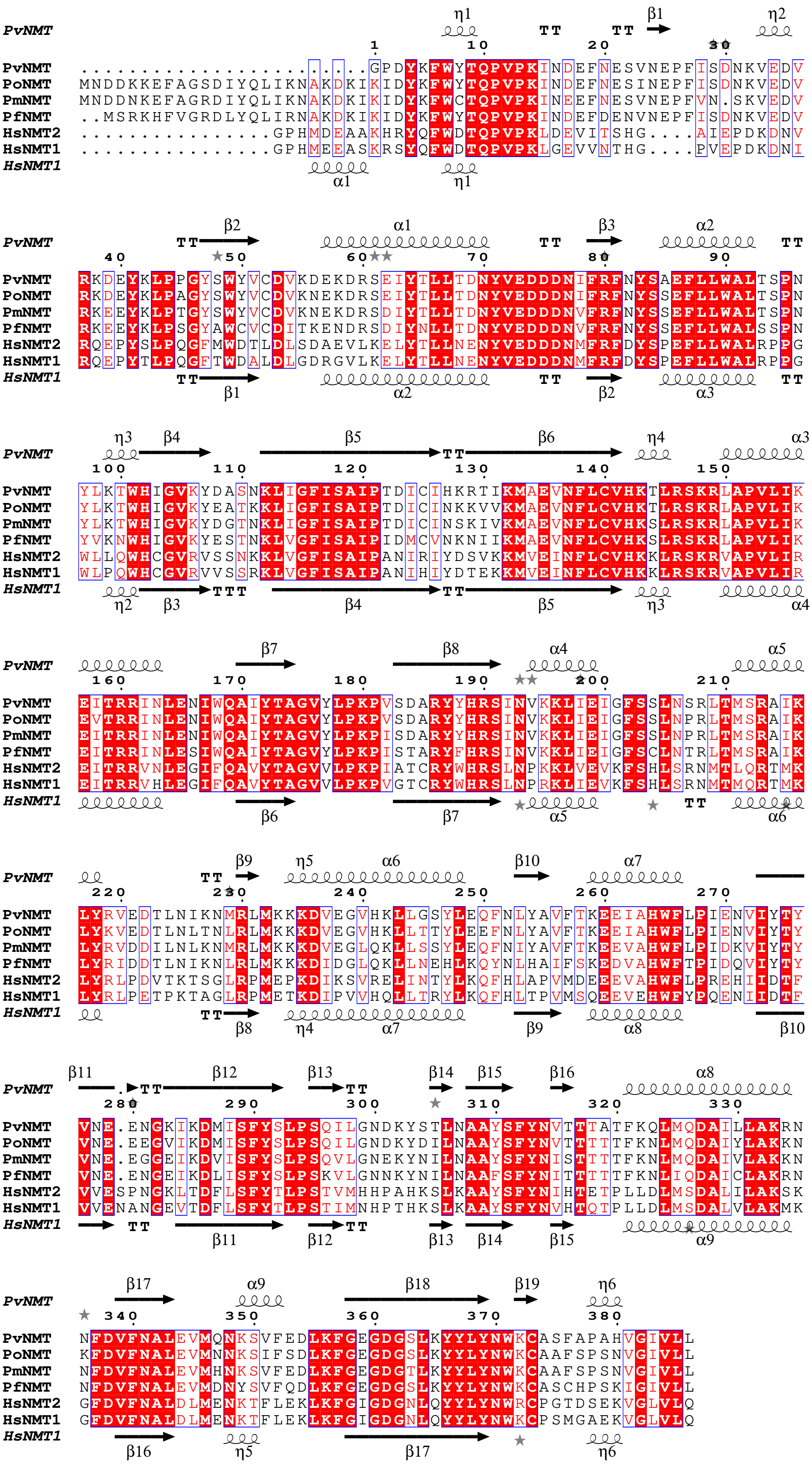
PvNMT ........................GPDYKFWYTQPVPKINDEFNESVNEPFISDNKVEDV
PoNMT MNDDKKEFAGSDIYQLIKNAKDKIKIDYKFWYTQPVPKINDEFNESINEPFISDNKVEDV
PmNMT MNDDNKEFAGRDIYQLIKNAKDKIKIDYKFWCTQPVPKINEEFNESVNEPFVN.SKVEDV
PfNMT ..MSRKHFVGRDLYQLIRNAKDKIKIDYKFWYTQPVPKINDEFDENVNEPFISDNKVEDV
HsNMT2 ................GPHMDEAAKHRYQFWDTQPVPKLDEVITSHG....AIEPDKDNV
HsNMT1 ................GPHMEEASKRSYQFWDTQPVPKLGEVVNTHG....PVEPDKDNI
PvNMT RKDEYKLPPGYSWYVCDVKDEKDRSEIYTLLTDNYVEDDDNIFRFNYSAEFLLWALTSPN
PoNMT RKDEYKLPAGYSWYVCDVKNEKDRSEIYTLLTDNYVEDDDNIFRFNYSSEFLLWALTSPN
PmNMT RKEEYKLPTGYSWYVCDVKNEKDRSDIYTLLTDNYVEDDDNVFRFNYSSEFLLWALTSPN
PfNMT RKEEYKLPSGYAWCVCDITKENDRSDIYNLLTDNYVEDDDNVFRFNYSSEFLLWALSSPN
HsNMT2 RQEPYSLPQGFMWDTLDLSDAEVLKELYTLLNENYVEDDDNMFRFDYSPEFLLWALRPPG
HsNMT1 RQEPYTLPQGFTWDALDLGDRGVLKELYTLLNENYVEDDDNMFRFDYSPEFLLWALRPPG
PvNMT YLKTWHIGVKYDASNKLIGFISAIPTDICIHKRTIKMAEVNFLCVHKTLRSKRLAPVLIK
PoNMT YLKTWHIGVKYEATKKLIGFISAIPTDICINKKVVKMAEVNFLCVHKTLRSKRLAPVLIK
PmNMT YLKTWHIGVKYDGTNKLIGFISAIPTDICINSKIVKMAEVNFLCVHKSLRSKRLAPVLIK
PfNMT YVKNWHIGVKYESTNKLVGFISAIPIDMCVNKNIIKMAEVNFLCVHKSLRSKRLAPVLIK
HsNMT2 WLLQWHCGVRVSSNKKLVGFISAIPANIRIYDSVKKMVEINFLCVHKKLRSKRVAPVLIR
HsNMT1 WLPQWHCGVRVVSSRKLVGFISAIPANIHIYDTEKKMVEINFLCVHKKLRSKRVAPVLIR
PvNMT EITRRINLENIWQAIYTAGVYLPKPVSDARYYHRSINVKKLIEIGFSSLNSRLTMSRAIK
PoNMT EVTRRINLENIWQAIYTAGVYLPKPVSDARYYHRSINVKKLIEIGFSSLNPRLTMSRAIK
PmNMT EITRRINLENIWQAIYTAGVYLPKPISDARYYHRSINVKKLIEIGFSSLNPRLTMSRAIK
PfNMT EITRRINLESIWQAIYTAGVYLPKPISTARYFHRSINVKKLIEIGFSCLNTRLTMSRAIK
HsNMT2 EITRRVNLEGIFQAVYTAGVVLPKPIATCRYWHRSLNPKKLVEVKFSHLSRNMTLQRTMK
HsNMT1 EITRRVHLEGIFQAVYTAGVVLPKPVGTCRYWHRSLNPRKLIEVKFSHLSRNMTMQRTMK
PvNMT LYRVEDTLNIKNMRLMKKKDVEGVHKLLGSYLEQFNLYAVFTKEEIAHWFLPIENVIYTY
PoNMT LYKVEDTLNLTNLRLMKKKDVEGVHKLLTTYLEEFNLYAVFTKEEIAHWFLPIENVIYTY
PmNMT LYRVDDILNLKNMRLMKKKDVEGLQKLLSSYLEQFNIYAVFTKEEVAHWFLPIDKVIYTY
PfNMT LYRIDDTLNIKNLRLMKKKDIDGLQKLLNEHLKQYNLHAIFSKEDVAHWFTPIDQVIYTY
HsNMT2 LYRLPDVTKTSGLRPMEPKDIKSVRELINTYLKQFHLAPVMDEEEVAHWFLPREHIIDTF
HsNMT1 LYRLPETPKTAGLRPMETKDIPVVHQLLTRYLKQFHLTPVMSQEEVEHWFYPQENIIDTF
PvNMT VNE.ENGKIKDMISFYSLPSQILGNDKYSTLNAAYSFYNVTTTATFKQLMQDAILLAKRN
PoNMT VNE.EEGVIKDMISFYSLPSQILGNDKYDILNAAYSFYNVTTTTTFKNLMQDAIYLAKKN
PmNMT VNE.EGGEIKDVISFYSLPSQVLGNEKYNILNAAYSFYNISTTTTFKNLMQDAIVLAKKN
PfNMT VNE.ENGEIKDLISFYSLPSKVLGNNKYNILNAAFSFYNITTTTTFKNLIQDAICLAKRN
HsNMT2 VVESPNGKLTDFLSFYTLPSTVMHHPAHKSLKAAYSFYNIHTETPLLDLMSDALILAKSK
HsNMT1 VVENANGEVTDFLSFYTLPSTIMNHPTHKSLKAAYSFYNVHTQTPLLDLMSDALVLAKMK
PvNMT NFDVFNALEVMQNKSVFEDLKFGEGDGSLKYYLYNWKCASFAPAHVGIVLL
PoNMT KFDVFNALEVMNNKSIFSDLKFGEGDGSLKYYLYNWKCAAFSPSNVGIVLL
PmNMT NFDVFNALEVMHNKSVFEDLKFGEGDGTLKYYLYNWKCAAFSPSNVGIVLL
PfNMT NFDVFNALEVMDNYSVFQDLKFGEGDGSLKYYLYNWKCASCHPSKIGIVLL
HsNMT2 GFDVFNALDLMENKTFLEKLKFGIGDGNLQYYLYNWRCPGTDSEKVGLVLQ
HsNMT1 GFDVFNALDLMENKTFLEKLKFGIGDGNLQYYLYNWKCPSMGAEKVGLVLQ

Table S1. Summar y Table from PDBeFold (http://www.ebi.ac.uk/msd-srv/ssm/) analysis

PDBe Fold v2.59. (src3) 14 Apr 2014 result file.

RESULT SUMMARY

##	Q-score	P-score	Z-score	RMSD	Nalgn	Nsse	Ngaps	Seq-%	Nmd	Nres-Q	Nsse-Q	Nres-T	Nsse-T	Query	Target
1	1	100.8	30.3	0.000	385	24	0	1	0	385	24	385	24	PDB 5v0w:A	PDB 5v0w:A
2	0.997	67.69	24.78	0.164	385	23	0	1	0	385	24	385	24	PDB 5v0w:A	PDB 5v0w:B
3	0.9959	62.08	23.71	0.193	385	23	0	1	0	385	24	385	24	PDB 5v0w:A	PDB 6mb1:A
4	0.9957	58.76	23.06	0.197	385	23	0	0.9974	0	385	24	385	24	PDB 5v0w:A	PDB 6mb0:A
5	0.9957	62.08	23.71	0.197	385	23	0	0.9974	0	385	24	385	24	PDB 5v0w:A	PDB 4ufv:A
6	0.9956	61.03	23.51	0.199	385	24	0	0.9974	0	385	24	385	24	PDB 5v0w:A	PDB 4b10:A
7	0.9955	62.38	23.77	0.202	385	24	0	0.9974	0	385	24	385	24	PDB 5v0w:A	PDB 5g1z:A
8	0.9951	60.08	23.32	0.211	385	23	0	0.9974	0	385	24	385	24	PDB 5v0w:A	PDB 4b11:A
9	0.995	60.63	23.43	0.212	385	23	0	1	0	385	24	385	24	PDB 5v0w:A	PDB 6mb1:B
10	0.9949	61.77	23.65	0.216	385	23	0	0.9974	0	385	24	385	24	PDB 5v0w:A	PDB 5o48:B
11	0.9949	60.08	23.32	0.216	385	23	0	0.9974	0	385	24	385	24	PDB 5v0w:A	PDB 4b13:A
12	0.9948	64.72	24.22	0.218	385	23	0	0.9974	0	385	24	385	25	PDB 5v0w:A	PDB 6mb0:B
13	0.9943	61.77	23.65	0.226	385	23	0	0.9974	0	385	24	385	24	PDB 5v0w:A	PDB 4ufw:B
14	0.9943	58.76	23.06	0.228	385	23	0	0.9974	0	385	24	385	25	PDB 5v0w:A	PDB 4ufw:A
15	0.9942	61.77	23.65	0.229	385	23	0	0.9974	0	385	24	385	24	PDB 5v0w:A	PDB 6maz:B
16	0.9942	58.25	22.96	0.229	385	22	0	0.9974	0	385	24	385	24	PDB 5v0w:A	PDB 4ufv:B
17	0.994	59.02	23.11	0.232	385	23	0	1	0	385	24	385	25	PDB 5v0w:A	PDB 6nxg:A
18	0.994	60.08	23.32	0.233	385	23	0	0.9974	0	385	24	385	24	PDB 5v0w:A	PDB 4b14:A
19	0.9938	56.88	22.63	0.180	384	23	0	1	0	385	24	384	24	PDB 5v0w:A	PDB 2ync:A
20	0.9933	59.28	23.17	0.247	385	23	0	0.9974	0	385	24	385	24	PDB 5v0w:A	PDB 6maz:A
21	0.9933	59.55	23.22	0.247	385	23	0	0.9974	0	385	24	385	25	PDB 5v0w:A	PDB 5g1z:B
22	0.9932	58.51	23.01	0.247	385	23	0	0.9974	0	385	24	385	24	PDB 5v0w:A	PDB 4b11:B
23	0.9932	61.48	23.6	0.248	385	23	0	0.9974	0	385	24	385	24	PDB 5v0w:A	PDB 4b12:B
24	0.9931	64.41	24.16	0.251	385	24	0	1	0	385	24	385	24	PDB 5v0w:A	PDB 5v0x:A
25	0.993	60.63	23.43	0.253	385	23	0	0.9974	0	385	24	385	24	PDB 5v0w:A	PDB 4b12:A
26	0.9927	58.76	23.06	0.258	385	23	0	0.9974	0	385	24	385	24	PDB 5v0w:A	PDB 5o48:A
27	0.9926	58.51	23.01	0.259	385	23	0	1	0	385	24	385	24	PDB 5v0w:A	PDB 6nxg:B
28	0.992	58.01	22.91	0.222	384	23	0	1	0	385	24	384	24	PDB 5v0w:A	PDB 4cae:B
29	0.9919	58.76	23.06	0.224	384	22	0	1	0	385	24	384	24	PDB 5v0w:A	PDB 4bbh:B
30	0.9918	61.48	23.6	0.225	384	23	0	1	0	385	24	384	25	PDB 5v0w:A	PDB 4caf:B
31	0.9916	61.48	23.6	0.229	384	23	0	1	0	385	24	384	24	PDB 5v0w:A	PDB 2yne:B
32	0.9916	60.08	23.32	0.276	385	23	0	0.9974	0	385	24	385	24	PDB 5v0w:A	PDB 5o4v:B
33	0.9916	63.22	23.93	0.277	385	24	0	0.9974	0	385	24	385	24	PDB 5v0w:A	PDB 4b14:B
34	0.9915	62.38	23.77	0.278	385	24	0	1	0	385	24	385	24	PDB 5v0w:A	PDB 5v0x:B
35	0.9915	55.13	22.27	0.278	385	22	0	0.9974	0	385	24	385	25	PDB 5v0w:A	PDB 4b10:B
36	0.9911	58.01	22.91	0.239	384	23	0	1	0	385	24	384	25	PDB 5v0w:A	PDB 4cae:A
37	0.991	59.81	23.27	0.286	385	23	0	0.9974	0	385	24	385	25	PDB 5v0w:A	PDB 4ufx:A
38	0.9909	60.63	23.43	0.243	384	23	0	1	0	385	24	384	24	PDB 5v0w:A	PDB 4bbh:A
39	0.9905	59.55	23.22	0.250	384	22	0	1	0	385	24	384	24	PDB 5v0w:A	PDB 2yne:A
40	0.9903	54.37	22.11	0.296	385	22	0	0.9974	0	385	24	385	24	PDB 5v0w:A	PDB 2ynd:B
41	0.9902	54.17	22.07	0.257	384	22	0	1	0	385	24	384	24	PDB 5v0w:A	PDB 2ync:B
42	0.9901	59.81	23.27	0.301	385	23	0	0.9974	0	385	24	385	24	PDB 5v0w:A	PDB 5o4v:A
43	0.99	61.57	23.61	0.301	385	24	0	0.9974	0	385	24	385	26	PDB 5v0w:A	PDB 4b13:B
44	0.99	57.77	22.86	0.259	384	22	0	1	0	385	24	384	24	PDB 5v0w:A	PDB 4caf:A
45	0.9899	56.74	22.6	0.303	385	22	0	0.9974	0	385	24	385	24	PDB 5v0w:A	PDB 2ynd:A
46	0.9898	62.38	23.77	0.304	385	23	0	0.9974	0	385	24	385	26	PDB 5v0w:A	PDB 4ufx:B
47	0.9884	70.68	25.32	0.286	384	24	0	1	0	385	24	384	24	PDB 5v0w:A	PDB 4c68:A
48	0.9881	59.28	23.17	0.290	384	23	0	1	0	385	24	384	24	PDB 5v0w:A	PDB 4a95:B
49	0.9863	59.28	23.17	0.318	384	23	0	1	0	385	24	384	24	PDB 5v0w:A	PDB 4a95:A
50	0.9855	60.08	23.32	0.247	382	23	1	0.9974	0	385	24	382	24	PDB 5v0w:A	PDB 6may:A
51	0.9851	61.84	23.67	0.335	384	23	0	1	0	385	24	384	25	PDB 5v0w:A	PDB 4c68:B
52	0.9836	55.6	22.43	0.387	385	23	0	0.9974	0	385	24	385	25	PDB 5v0w:A	PDB 5g22:B
53	0.9824	62.38	23.77	0.371	384	23	0	0.9974	0	385	24	384	25	PDB 5v0w:A	PDB 6may:C
54	0.9818	56.03	22.51	0.408	385	23	0	0.9974	0	385	24	385	24	PDB 5v0w:A	PDB 5g22:A
55	0.9535	57.09	22.73	0.517	378	23	1	1	0	385	24	378	24	PDB 5v0w:A	PDB 6b1l:A
56	0.9523	56.17	22.6	0.327	372	22	1	0.9892	0	385	24	373	23	PDB 5v0w:A	PDB 6maz:C
57	0.9508	51.21	22.65	0.312	372	21	2	0.9892	0	385	24	374	25	PDB 5v0w:A	PDB 6mb0:C
58	0.9503	57.19	22.81	0.319	370	20	1	1	0	385	24	370	23	PDB 5v0w:A	PDB 4c68:C
59	0.9502	55.44	22.45	0.320	371	22	1	0.9919	0	385	24	372	24	PDB 5v0w:A	PDB 4ufv:C
60	0.9495	52.77	21.84	0.428	375	22	1	0.984	0	385	24	377	24	PDB 5v0w:A	PDB 5v0w:C
61	0.9482	52.59	21.8	0.414	373	21	1	0.9839	0	385	24	374	24	PDB 5v0w:A	PDB 4b10:C
62	0.9471	55.67	22.5	0.288	368	22	1	1	0	385	24	368	23	PDB 5v0w:A	PDB 4b12:C
63	0.9471	57.72	22.92	0.290	371	22	2	0.9946	0	385	24	374	23	PDB 5v0w:A	PDB 6mb1:C
64	0.9469	54.98	22.36	0.366	371	21	1	0.9919	0	385	24	372	24	PDB 5v0w:A	PDB 4ufx:C
65	0.9464	55.44	22.45	0.339	371	22	2	0.9946	0	385	24	373	23	PDB 5v0w:A	PDB 5v0x:C
66	0.9453	57.01	22.77	0.317	368	22	1	0.9973	0	385	24	368	23	PDB 5v0w:A	PDB 4b13:C
67	0.9451	53.72	22.1	0.279	367	20	1	1	0	385	24	367	23	PDB 5v0w:A	PDB 4bbh:C
68	0.945	50.64	21.32	0.391	370	20	1	1	0	385	24	370	24	PDB 5v0w:A	PDB 5g1z:C
69	0.9449	53.92	22.14	0.360	371	21	2	0.9919	0	385	24	373	23	PDB 5v0w:A	PDB 4caf:C
70	0.9445	55.67	22.5	0.329	368	20	1	1	0	385	24	368	23	PDB 5v0w:A	PDB 5o48:C
71	0.9445	54.55	22.27	0.289	367	21	1	1	0	385	24	367	23	PDB 5v0w:A	PDB 2yne:C
72	0.944	55.21	22.41	0.403	370	22	1	0.9973	0	385	24	370	23	PDB 5v0w:A	PDB 2ync:C
73	0.9439	55.44	22.45	0.299	367	21	1	1	0	385	24	367	23	PDB 5v0w:A	PDB 4cae:C
74	0.9433	55.92	22.55	0.347	368	21	1	1	0	385	24	368	23	PDB 5v0w:A	PDB 2ynd:C
75	0.9432	55.44	22.45	0.310	367	20	1	1	0	385	24	367	23	PDB 5v0w:A	PDB 4ufw:C
76	0.9428	56.35	22.64	0.315	367	21	1	1	0	385	24	367	23	PDB 5v0w:A	PDB 4b14:C
77	0.9427	51.07	21.48	0.419	372	22	2	0.9866	0	385	24	374	25	PDB 5v0w:A	PDB 4b11:C
78	0.9417	55.44	22.45	0.429	370	22	1	0.9973	0	385	24	370	23	PDB 5v0w:A	PDB 6may:B
79	0.9398	57.19	22.81	0.392	368	20	1	1	0	385	24	368	23	PDB 5v0w:A	PDB 5o4v:C
80	0.9384	53.52	22.06	0.342	366	21	1	1	0	385	24	366	23	PDB 5v0w:A	PDB 4a95:C
81	0.9319	54.13	22.18	0.454	368	21	1	1	0	385	24	369	24	PDB 5v0w:A	PDB 5g22:C
82	0.927	49.94	21.24	0.428	371	21	2	0.9892	0	385	24	378	24	PDB 5v0w:A	PDB 6nxg:C
83	0.9107	47.65	20.67	0.808	379	21	4	0.5409	0	385	24	382	24	PDB 5v0w:A	PDB 6f56:C
84	0.9091	49.51	21.18	0.818	379	22	4	0.5488	0	385	24	382	25	PDB 5v0w:A	PDB 5o6h:A
85	0.9087	50	21.29	0.821	379	22	4	0.5488	0	385	24	382	25	PDB 5v0w:A	PDB 5o6j:A
86	0.9087	53.06	21.94	0.821	378	22	4	0.5503	0	385	24	380	25	PDB 5v0w:A	PDB 3iu1:A
87	0.9081	49.84	21.25	0.825	379	22	4	0.5488	0	385	24	382	25	PDB 5v0w:A	PDB 3jtk:A
88	0.9073	48.25	20.87	0.845	379	21	4	0.5488	0	385	24	381	23	PDB 5v0w:A	PDB 5o9s:B
89	0.9066	51.56	21.62	0.834	379	22	4	0.5488	0	385	24	382	24	PDB 5v0w:A	PDB 6fz5:A
90	0.9061	54.89	22.32	0.837	378	22	4	0.5503	0	385	24	380	24	PDB 5v0w:A	PDB 6fz5:B
91	0.9061	49.19	21.11	0.837	379	22	4	0.5488	0	385	24	382	25	PDB 5v0w:A	PDB 4c2y:A
92	0.9059	49.84	21.25	0.807	376	22	4	0.5505	0	385	24	378	24	PDB 5v0w:A	PDB 6fz3:B
93	0.9058	54.05	22.14	0.839	379	22	4	0.5409	0	385	24	382	24	PDB 5v0w:A	PDB 6f56:A
94	0.9044	56.04	22.55	0.848	379	22	4	0.5488	0	385	24	382	24	PDB 5v0w:A	PDB 3iwe:A
95	0.9037	50.09	21.31	0.822	377	23	4	0.5517	0	385	24	380	24	PDB 5v0w:A	PDB 5uut:B
96	0.9024	52.07	21.73	0.831	378	23	4	0.5503	0	385	24	382	25	PDB 5v0w:A	PDB 3iu2:A

97	0.9022	51.9	21.69	0.862	379	23	4	0.5488	0	385	24	382	26	PDB 5v0w:A	PDB 4c2z:A
98	0.9018	49.84	21.25	0.864	379	22	4	0.5488	0	385	24	382	24	PDB 5v0w:A	PDB 5mu6:A
99	0.8961	49.35	21.15	0.808	372	22	4	0.5538	0	385	24	374	24	PDB 5v0w:A	PDB 6fz2:B
100	0.8956	52.42	21.8	0.827	373	23	4	0.5523	0	385	24	375	24	PDB 5v0w:A	PDB 5npq:B
101	0.8949	44.54	20.04	0.832	374	20	5	0.5561	0	385	24	377	23	PDB 5v0w:A	PDB 6pav:A
102	0.8948	49.35	21.15	0.819	379	22	4	0.5303	0	385	24	388	24	PDB 5v0w:A	PDB 4c2x:A
103	0.8912	48.87	21.04	0.871	375	22	5	0.5547	0	385	24	378	24	PDB 5v0w:A	PDB 5o9s:A
104	0.8907	50.67	21.43	0.861	379	22	4	0.5488	0	385	24	387	24	PDB 5v0w:A	PDB 6fz3:A
105	0.8896	49.19	21.11	0.836	374	22	5	0.5588	0	385	24	379	24	PDB 5v0w:A	PDB 6pav:B
106	0.8894	50.5	21.4	0.821	371	22	5	0.558	0	385	24	374	24	PDB 5v0w:A	PDB 6fz2:A
107	0.8883	51.38	21.58	0.817	379	22	4	0.5488	0	385	24	391	25	PDB 5v0w:A	PDB 7owr:B
108	0.8878	51.37	21.48	0.805	379	22	4	0.5488	0	385	24	392	26	PDB 5v0w:A	PDB 7owm:A
109	0.8863	53.89	22.11	0.798	377	23	4	0.5517	0	385	24	389	25	PDB 5v0w:A	PDB 6sjz:B
110	0.886	45.03	20.19	0.904	377	22	5	0.5517	0	385	24	382	25	PDB 5v0w:A	PDB 4c2y:B
111	0.8858	48.55	20.97	0.905	377	22	5	0.5517	0	385	24	382	25	PDB 5v0w:A	PDB 4c2z:B
112	0.8857	46.15	20.44	0.905	375	22	5	0.5547	0	385	24	378	25	PDB 5v0w:A	PDB 3jtk:B
113	0.885	50.9	21.48	0.824	379	23	4	0.5488	0	385	24	392	25	PDB 5v0w:A	PDB 6qrm:A
114	0.885	52.59	21.84	0.788	374	23	4	0.5535	0	385	24	384	24	PDB 5v0w:A	PDB 6ehj:B
115	0.885	51.56	21.62	0.809	379	23	4	0.5488	0	385	24	393	24	PDB 5v0w:A	PDB 7own:A
116	0.8848	52.95	21.91	0.825	379	23	4	0.5488	0	385	24	392	24	PDB 5v0w:A	PDB 7owo:A
117	0.8846	47.32	20.7	0.837	372	22	6	0.5538	0	385	24	377	24	PDB 5v0w:A	PDB 5mu6:B
118	0.8842	53.7	22.07	0.829	379	23	4	0.5488	0	385	24	392	25	PDB 5v0w:A	PDB 5o9t:A
119	0.8836	46.15	20.44	0.919	377	22	5	0.5517	0	385	24	382	25	PDB 5v0w:A	PDB 3iu1:B
120	0.8835	49.19	21.11	0.919	377	22	5	0.5517	0	385	24	382	26	PDB 5v0w:A	PDB 5o6h:B
121	0.883	52.12	21.74	0.837	379	22	4	0.5488	0	385	24	392	26	PDB 5v0w:A	PDB 6sk2:A
122	0.8828	56.06	22.55	0.823	379	23	4	0.5488	0	385	24	393	25	PDB 5v0w:A	PDB 5o9u:A
123	0.8817	49.19	21.11	0.830	378	22	4	0.5503	0	385	24	391	25	PDB 5v0w:A	PDB 7owu:A
124	0.8812	51.2	21.54	0.817	377	22	4	0.5491	0	385	24	390	25	PDB 5v0w:A	PDB 6skj:B
125	0.881	51.23	21.55	0.836	379	23	4	0.5488	0	385	24	393	25	PDB 5v0w:A	PDB 5o9v:A
126	0.8806	47.02	20.64	0.964	378	22	5	0.545	0	385	24	382	24	PDB 5v0w:A	PDB 6f56:B
127	0.8793	49.51	21.18	0.902	376	22	5	0.5532	0	385	24	383	26	PDB 5v0w:A	PDB 5o6j:B
128	0.8782	48.87	21.04	0.937	376	22	5	0.5532	0	385	24	381	24	PDB 5v0w:A	PDB 3iwe:B
129	0.8779	43.75	19.79	0.909	371	20	5	0.5606	0	385	24	373	23	PDB 5v0w:A	PDB 5uut:A
130	0.8772	51.74	21.66	0.846	379	22	4	0.5488	0	385	24	394	26	PDB 5v0w:A	PDB 7owp:A
131	0.8759	54.85	22.27	0.840	379	22	4	0.5488	0	385	24	395	25	PDB 5v0w:A	PDB 7owu:B
132	0.8759	50.33	21.36	0.978	376	22	5	0.5532	0	385	24	379	25	PDB 5v0w:A	PDB 3iu2:B
133	0.8753	43.32	20.63	0.901	379	21	4	0.5488	0	385	24	391	24	PDB 5v0w:A	PDB 7owq:A
134	0.8746	45.45	20.28	0.999	378	22	4	0.5423	0	385	24	382	24	PDB 5v0w:A	PDB 6f56:D
135	0.8743	49.03	21.08	0.846	375	22	5	0.5547	0	385	24	387	25	PDB 5v0w:A	PDB 7owm:B
136	0.8742	48.2	21.66	0.831	375	23	5	0.552	0	385	24	388	25	PDB 5v0w:A	PDB 7owr:A
137	0.8717	46.3	20.47	0.849	376	22	5	0.5532	0	385	24	390	25	PDB 5v0w:A	PDB 6sk2:B
138	0.8711	51.2	21.54	0.840	377	22	5	0.5517	0	385	24	393	25	PDB 5v0w:A	PDB 5o9u:B
139	0.871	48.87	21.04	0.838	375	22	5	0.5547	0	385	24	389	25	PDB 5v0w:A	PDB 7owo:B
140	0.8689	50.85	21.47	0.883	377	22	5	0.5517	0	385	24	391	25	PDB 5v0w:A	PDB 6sjz:A
141	0.8685	49.35	21.15	0.885	377	22	5	0.5517	0	385	24	391	25	PDB 5v0w:A	PDB 6qrm:B
142	0.8685	49.67	21.22	0.857	377	22	5	0.5517	0	385	24	393	25	PDB 5v0w:A	PDB 5o9t:B
143	0.8679	48.56	20.87	0.875	377	21	5	0.5517	0	385	24	392	25	PDB 5v0w:A	PDB 7owp:B
144	0.8675	50	21.29	0.860	374	22	5	0.5508	0	385	24	387	25	PDB 5v0w:A	PDB 6sk8:B
145	0.8637	51.2	21.54	0.888	377	22	5	0.5517	0	385	24	393	25	PDB 5v0w:A	PDB 5o9v:B
146	0.8636	47.06	20.54	0.852	369	21	6	0.5556	0	385	24	379	23	PDB 5v0w:A	PDB 6ehj:A
147	0.861	47.02	20.64	0.842	372	22	5	0.5565	0	385	24	387	25	PDB 5v0w:A	PDB 6skj:A
148	0.861	50.9	21.48	0.890	376	23	4	0.5479	0	385	24	392	25	PDB 5v0w:A	PDB 6sk8:A
149	0.8606	42.36	20.31	0.890	374	22	5	0.5561	0	385	24	388	24	PDB 5v0w:A	PDB 7owq:B
150	0.8587	50.9	21.48	0.887	373	23	5	0.5523	0	385	24	387	25	PDB 5v0w:A	PDB 6sk3:A
151	0.8575	51.02	21.51	0.927	377	22	5	0.5517	0	385	24	393	25	PDB 5v0w:A	PDB 7own:B
152	0.8554	47.47	20.7	0.805	357	21	4	0.5294	0	385	24	361	24	PDB 5v0w:A	PDB 6pau:A
153	0.8479	50.57	21.41	0.914	373	23	5	0.5523	0	385	24	390	25	PDB 5v0w:A	PDB 6sk3:B
154	0.8359	45.81	20.32	0.855	350	21	2	0.5629	0	385	24	352	23	PDB 5v0w:A	PDB 7rk3:A
155	0.8316	44.09	19.97	0.931	353	21	6	0.5552	0	385	24	355	23	PDB 5v0w:A	PDB 5npq:A
156	0.8253	45.73	20.44	0.981	376	20	5	0.4388	0	385	24	402	27	PDB 5v0w:A	PDB 5ag6:A
157	0.8213	45.73	20.44	1.026	381	21	5	0.4331	0	385	24	411	29	PDB 5v0w:A	PDB 6qdg:A
158	0.8135	42.72	19.76	0.980	377	21	4	0.4377	0	385	24	410	28	PDB 5v0w:A	PDB 8fi5:A
159	0.8132	45.3	20.35	0.997	378	21	7	0.4365	0	385	24	411	28	PDB 5v0w:A	PDB 4a30:A
160	0.8131	45.44	20.38	1.024	379	21	7	0.4354	0	385	24	411	29	PDB 5v0w:A	PDB 4c7h:A
161	0.8123	42.23	20.25	1.002	378	21	7	0.4365	0	385	24	411	28	PDB 5v0w:A	PDB 4a32:A
162	0.8122	44.19	20.09	1.054	380	20	6	0.4342	0	385	24	411	28	PDB 5v0w:A	PDB 8fi6:A
163	0.8119	45.65	20.38	1.031	379	21	7	0.4354	0	385	24	411	30	PDB 5v0w:A	PDB 5g21:A
164	0.8118	45.87	20.47	0.979	377	20	7	0.4377	0	385	24	411	28	PDB 5v0w:A	PDB 4ucp:A
165	0.8113	45.44	20.38	1.034	379	20	7	0.4354	0	385	24	411	28	PDB 5v0w:A	PDB 6eu5:A
166	0.8106	45.73	20.44	1.013	378	21	7	0.4365	0	385	24	411	27	PDB 5v0w:A	PDB 5age:A
167	0.8094	45.3	20.35	1.071	380	21	6	0.4342	0	385	24	411	30	PDB 5v0w:A	PDB 6qdf:A
168	0.8075	45.16	20.31	0.979	376	20	6	0.4388	0	385	24	411	28	PDB 5v0w:A	PDB 5ag4:A
169	0.8059	46.01	20.51	1.041	378	21	7	0.4365	0	385	24	411	28	PDB 5v0w:A	PDB 6qdh:A
170	0.8048	44.88	20.25	1.048	378	21	8	0.4339	0	385	24	411	28	PDB 5v0w:A	PDB 2wsa:A
171	0.8043	45.73	20.44	1.051	378	21	6	0.4392	0	385	24	411	28	PDB 5v0w:A	PDB 4cgm:A
172	0.8027	44.46	20.16	1.035	377	20	5	0.4377	0	385	24	411	28	PDB 5v0w:A	PDB 4ucn:A
173	0.8025	44.45	20.02	1.061	378	22	8	0.4339	0	385	24	411	29	PDB 5v0w:A	PDB 4a2z:A
174	0.8023	43.11	19.85	1.136	381	20	5	0.4331	0	385	24	411	28	PDB 5v0w:A	PDB 8fi4:A
175	0.8022	44.32	20.13	1.088	379	21	7	0.4327	0	385	24	411	28	PDB 5v0w:A	PDB 4a31:A
176	0.8017	44.18	19.96	1.066	378	22	8	0.4339	0	385	24	411	29	PDB 5v0w:A	PDB 4a33:A
177	0.8011	44.88	20.25	0.992	375	20	7	0.4347	0	385	24	411	28	PDB 5v0w:A	PDB 6gns:A
178	0.8007	43.11	19.85	1.072	378	21	7	0.4365	0	385	24	411	28	PDB 5v0w:A	PDB 6ewf:A
179	0.7995	44.46	20.16	1.080	378	21	7	0.4365	0	385	24	411	29	PDB 5v0w:A	PDB 6qdb:A
180	0.798	43.05	19.83	1.113	379	21	7	0.4327	0	385	24	411	30	PDB 5v0w:A	PDB 4cyq:A
181	0.7976	42.66	19.74	1.079	378	22	8	0.4339	0	385	24	412	28	PDB 5v0w:A	PDB 5ag7:A
182	0.7975	44.46	20.16	1.066	377	21	5	0.4377	0	385	24	411	30	PDB 5v0w:A	PDB 4cgp:A
183	0.7968	43.91	20.03	1.095	378	21	7	0.4339	0	385	24	411	30	PDB 5v0w:A	PDB 5g20:A
184	0.7964	45.12	20.17	1.072	377	21	8	0.435	0	385	24	411	28	PDB 5v0w:A	PDB 5ag5:A
185	0.7962	44.05	20.06	1.022	375	20	8	0.432	0	385	24	411	28	PDB 5v0w:A	PDB 6gnu:A
186	0.7961	43.18	19.86	1.123	379	22	7	0.4327	0	385	24	411	30	PDB 5v0w:A	PDB 6qdd:A
187	0.7957	44.6	20.19	1.077	377	21	5	0.4377	0	385	24	411	29	PDB 5v0w:A	PDB 6qd9:A
188	0.7951	44.6	20.19	1.080	377	21	5	0.4377	0	385	24	411	30	PDB 5v0w:A	PDB 6qdc:A
189	0.7942	43.71	19.99	1.086	377	21	8	0.435	0	385	24	411	30	PDB 5v0w:A	PDB 4cgn:A
190	0.794	44.6	20.19	1.062	376	21	6	0.4388	0	385	24	411	30	PDB 5v0w:A	PDB 6qda:A
191	0.7928	44.19	20.09	1.069	376	21	6	0.4388	0	385	24	411	29	PDB 5v0w:A	PDB 4cyn:A
192	0.7926	43.18	19.65	1.120	378	22	8	0.4339	0	385	24	411	31	PDB 5v0w:A	PDB 5a27:A
193	0.7925	45.37	20.16	1.071	376	21	6	0.4388	0	385	24	411	30	PDB 5v0w:A	PDB 6qde:A
194	0.7924	42.23	20.25	1.046	375	20	7	0.4347	0	385	24	411	28	PDB 5v0w:A	PDB 6gnt:A
195	0.7915	42.92	19.8	1.102	377	22	8	0.435	0	385	24	411	28	PDB 5v0w:A	PDB 4cgl:A
196	0.7906	44.3	20.11	1.107	377	21	8	0.4324	0	385	24	411	29	PDB 5v0w:A	PDB 6gnh:A
197	0.7877	36.93	18.96	1.074	375	20	9	0.432	0	385	24	411	27	PDB 5v0w:A	PDB 6gnv:A
198	0.7863	42.53	19.71	1.107	376	21	6	0.4362	0	385	24	411	30	PDB 5v0w:A	PDB 4cyp:A

199	0.7847	41.36	19.34	1.092	375	21	7	0.4373	0	385	24	411	29	PDB 5v0w:A	PDB 4ucm:A
200	0.7797	38.3	18.64	1.022	376	22	6	0.4548	0	385	24	422	25	PDB 5v0w:A	PDB 2nmt:A
201	0.7797	43.92	19.89	1.122	375	22	7	0.4373	0	385	24	411	31	PDB 5v0w:A	PDB 4cgo:A
202	0.7772	41.51	19.48	1.184	377	22	8	0.435	0	385	24	411	28	PDB 5v0w:A	PDB 3h5z:A
203	0.7658	43.28	19.79	0.990	358	21	6	0.4721	0	385	24	392	25	PDB 5v0w:A	PDB 4uwj:A
204	0.7653	43.67	19.88	0.994	358	21	6	0.4721	0	385	24	392	25	PDB 5v0w:A	PDB 5t6h:A
205	0.7633	43.05	19.69	1.006	358	20	6	0.4721	0	385	24	392	25	PDB 5v0w:A	PDB 5t5u:A
206	0.7608	44.48	20.06	0.974	354	21	6	0.4718	0	385	24	387	25	PDB 5v0w:A	PDB 4cax:A
207	0.7603	45.73	20.35	1.026	358	22	6	0.4721	0	385	24	392	26	PDB 5v0w:A	PDB 5t6e:A
208	0.7579	46.44	20.51	1.014	357	22	5	0.4734	0	385	24	392	26	PDB 5v0w:A	PDB 5t6c:A
209	0.7569	36.81	18.34	1.277	372	21	5	0.4247	0	385	24	402	28	PDB 5v0w:A	PDB 2wuu:A
210	0.7553	42.25	19.55	1.070	358	21	6	0.4721	0	385	24	391	25	PDB 5v0w:A	PDB 4uwi:A
211	0.7545	42.63	19.64	1.061	357	21	5	0.4734	0	385	24	390	24	PDB 5v0w:A	PDB 4caw:A
212	0.7428	37.04	18.4	1.270	375	21	8	0.4187	0	385	24	417	28	PDB 5v0w:A	PDB 6omk:A
213	0.7421	42.62	19.59	1.177	373	22	9	0.4504	0	385	24	422	25	PDB 5v0w:A	PDB 1iic:B
214	0.7326	39.74	18.84	1.210	372	21	9	0.4462	0	385	24	422	24	PDB 5v0w:A	PDB 1iid:A
215	0.7292	43.16	19.96	1.166	371	23	7	0.4582	0	385	24	426	28	PDB 5v0w:A	PDB 8fbm:A
216	0.7275	43.1	19.99	1.245	374	23	6	0.4572	0	385	24	426	30	PDB 5v0w:A	PDB 8fbm:B
217	0.7222	42.11	19.48	1.178	368	22	8	0.4511	0	385	24	422	26	PDB 5v0w:A	PDB 1iic:A
218	0.7169	39.55	18.9	1.246	358	21	6	0.4609	0	385	24	396	26	PDB 5v0w:A	PDB 4qbj:A
219	0.7127	38.3	18.56	1.243	355	20	7	0.4704	0	385	24	392	25	PDB 5v0w:A	PDB 4cav:A
220	0.703	28.59	16.26	1.152	368	21	8	0.4538	0	385	24	436	26	PDB 5v0w:A	PDB 2p6f:D
221	0.7025	35.27	17.95	1.156	368	21	8	0.4538	0	385	24	436	25	PDB 5v0w:A	PDB 2p6f:A
222	0.7018	41.11	19.24	1.160	368	23	8	0.4538	0	385	24	436	27	PDB 5v0w:A	PDB 2p6f:B
223	0.7007	37.35	18.32	1.214	370	22	8	0.4486	0	385	24	436	26	PDB 5v0w:A	PDB 2p6f:E
224	0.6989	41.07	19.27	1.165	367	23	8	0.4523	0	385	24	435	25	PDB 5v0w:A	PDB 2p6g:D
225	0.6989	36.68	18.2	1.140	366	21	9	0.4508	0	385	24	435	25	PDB 5v0w:A	PDB 2p6g:A
226	0.6975	37.86	18.49	1.197	368	22	8	0.4484	0	385	24	435	26	PDB 5v0w:A	PDB 2p6g:F
227	0.6951	40.5	19.09	1.212	368	23	8	0.4484	0	385	24	435	26	PDB 5v0w:A	PDB 2p6g:B
228	0.6947	39.67	18.89	1.145	368	22	8	0.4538	0	385	24	442	26	PDB 5v0w:A	PDB 2p6e:A
229	0.6943	36.35	18.12	1.112	367	21	9	0.455	0	385	24	443	25	PDB 5v0w:A	PDB 2p6e:D
230	0.6925	31.98	17.73	1.132	364	20	9	0.4505	0	385	24	435	22	PDB 5v0w:A	PDB 2p6g:E
231	0.6913	34.62	17.68	1.206	368	21	8	0.4484	0	385	24	438	25	PDB 5v0w:A	PDB 2p6e:F
232	0.691	36.83	18.24	1.204	367	21	10	0.4605	0	385	24	436	26	PDB 5v0w:A	PDB 2p6f:C
233	0.6909	39.55	18.86	1.134	367	22	8	0.4496	0	385	24	443	26	PDB 5v0w:A	PDB 2p6e:E
234	0.6904	31.94	16.84	1.218	367	20	8	0.4496	0	385	24	435	26	PDB 5v0w:A	PDB 2p6g:C
235	0.6817	40.51	19.1	1.121	364	22	9	0.456	0	385	24	443	26	PDB 5v0w:A	PDB 2p6e:B
236	0.6804	36.46	18.14	1.197	368	21	8	0.4538	0	385	24	446	25	PDB 5v0w:A	PDB 2p6e:C
237	0.6783	29.11	16.19	1.200	363	19	9	0.4573	0	385	24	435	22	PDB 5v0w:A	PDB 2p6f:F
238	0.6685	31.5	16.87	1.519	356	22	9	0.4298	0	385	24	392	24	PDB 5v0w:A	PDB 1nmt:A
239	0.6681	32.34	17.08	1.521	356	21	9	0.4242	0	385	24	392	24	PDB 5v0w:A	PDB 1nmt:B
240	0.6587	29.8	16.41	1.560	354	22	8	0.4322	0	385	24	389	25	PDB 5v0w:A	PDB 1nmt:C
241	0.655	34.68	18.31	1.130	336	20	7	0.4494	0	385	24	392	24	PDB 5v0w:A	PDB 1iyk:B
242	0.6545	35.54	18.56	1.107	335	20	8	0.4478	0	385	24	392	24	PDB 5v0w:A	PDB 1iyk:A
243	0.6348	27.66	15.73	1.575	350	20	10	0.46	0	385	24	393	26	PDB 5v0w:A	PDB 8hbs:A
244	0.6199	29.53	16.17	1.406	333	19	10	0.4414	0	385	24	381	22	PDB 5v0w:A	PDB 1iyl:C
245	0.6138	31.47	16.8	1.437	334	20	9	0.4401	0	385	24	384	23	PDB 5v0w:A	PDB 1iyl:A
246	0.6087	25.37	15.08	1.463	332	19	11	0.4398	0	385	24	380	23	PDB 5v0w:A	PDB 1iyl:B
